# Supplementary material for: The causal role of circulating inflammatory markers in osteoporosis: a bidirectional Mendelian randomized study
Source: Front Immunol. 2024 Jul 18;15:1412298. doi: 10.3389/fimmu.2024.1412298 (PMC11291241; doi:10.3389/fimmu.2024.1412298)
Supplement: Supplementary file 1 [file DataSheet_1.zip › supplementary material/CIM-OSTEOPOROSIS /GCST90274825.csv_leave_one_out.pdf]

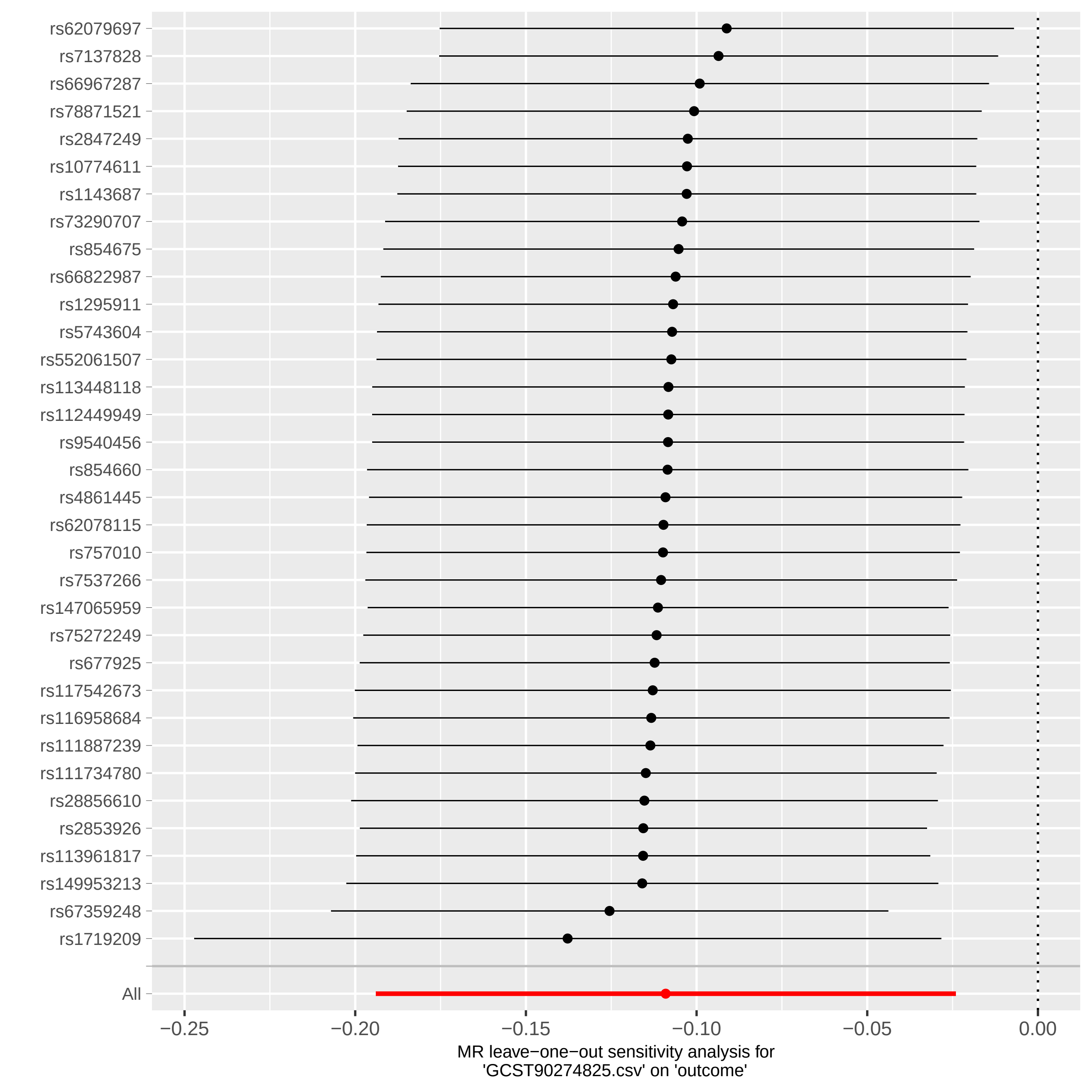
rs62079697
rs7137828
rs66967287
rs78871521
rs2847249
rs10774611
rs1143687
rs73290707
rs854675
rs66822987
rs1295911
rs5743604
rs552061507
rs113448118
rs112449949
rs9540456
rs854660
rs4861445
rs62078115
rs757010
rs7537266
rs147065959
rs75272249
rs677925
rs117542673
rs116958684
rs111887239
rs111734780
rs28856610
rs2853926
rs113961817
rs149953213
rs67359248
rs1719209
All
−0.25
−0.20
−0.15
−0.10
−0.05
0.00
MR leave−one−out sensitivity analysis for
'GCST90274825.csv' on 'outcome'
